# Impact of technological innovation on corporate leverage in China: The moderating role of policy incentives and market competition

**DOI:** 10.3389/fpsyg.2022.1068375

**Published:** 2022-12-01

**Authors:** Lin Ren, Dan Liu, Deping Xiong

**Affiliations:** ^1^School of Finance, Yunnan University of Finance and Economics, Kunming, China; ^2^School of Economics, Yunnan University of Finance and Economics, Kunming, China

**Keywords:** technological innovation, corporate leverage, policy incentives, market competition, China

## Abstract

Due to its capital-related nature, corporate leverage is highly exposed to financial risk, and optimizing corporate leverage is an effective method of mitigating financial risk to maximize corporate value. We use a two-way fixed effects model to examine the impact of technological innovation on corporate leverage using panel data of A-share listed companies in the Chinese manufacturing sector from 2012 to 2020. The results show that technological innovation and corporate leverage exhibit significant heterogeneity in cross-sectional, spatial and temporal dimensions. By further distinguishing between the effects of policy incentives and market competition, we find that the former exerts an “investment crowding out” effect and the latter an “innovation spillover” effect. These factors mitigate the negative relationship between technological innovation and corporate leverage. In general, this study provides empirical evidence for the rational allocation of resources by the Chinese government, the development of innovation capabilities, and the adjustment of leverage by firms from various regions.

## Introduction

As financial markets have developed rapidly, leveraged operations and expansion have become a global trend among corporations. For example, at one point, China’s non-financial corporate sector leverage ratio had risen to 272.5%, exceeding not only emerging economies but also developed economies such as the United States, Europe, and Japan. In a market economy, it makes sense for businesses to use external funding for operations and expansion. However, issues such as diminishing marginal benefits of corporate investment increased risk of default, and erosion of corporate performance due to over-leverage have become insurmountable business obstacles for businesses (Campello, 2006; Berk et al., 2010; Reinhart and Rogoff, 2010; Schularick and Taylor, 2012). Consequently, corporate leverage adjustment is widely adopted and utilized.

Following the theory of trade-offs, corporate leverage adjustment assists firms in coping with various risks arising from uncertainty and maximizes corporate value by achieving the desired capital structure. The empirical study demonstrates that firm heterogeneity and asymmetry result in different leverage adjustment costs at different leverage levels. The leverage effect is only triggered when firms’ profitability is sufficient to cover the adjustment costs (Aivazian et al., 2005; Galvao and Montes-Rojas, 2010). Consequently, corporate leverage adjustment is viable for companies to deal with uncertainty and attain their desired capital structure. Additional research into corporate leverage factors is necessary to achieve sustainable corporate growth.

Schumpeter’s concepts of “creative destruction” and “creative accumulation” concerning corporate innovation hold that innovation can be used as a new production function by altering the original conditions of production or by adding new factors of production to form a new system, thereby becoming a force for sustained economic growth. Moreover, according to research, technological innovation determines a company’s market value, comparative advantage, and return on investment, which is crucial for competitiveness, survival, and sustainable development (Myers, 1977; Porter, 1992). Therefore, technological innovation presents an opportunity for corporate leverage.

In China, the pattern of economic growth is shifting from factor-driven to technology-driven, and technological innovation has emerged as a determining factor for high-quality economic development. In the 14th 5-Year Plan for National Economic and Social Development and the 2035 Vision Plan (effective 2021), the Chinese government has proposed to improve the national innovation system and promote the concentration of various innovation factors in Chinese enterprises. At the firm level, innovative firms are the foundation for developing an innovative nation, and Chinese firms must enhance their market competitiveness by developing innovative products and services (Lee et al., 2021). On the one hand, they must manage their financial risks and continually optimize their leverage to maximize their company’s value. Notably, as Chinese firms have gradually shifted their innovation financing from a high reliance on foreign capital and international trade to domestic investment and government-subsidized innovation, this shift has had a unique effect on the relationship between technological innovation and firm leverage in Chinese firms. China is worth studying as an example of significant market failures and government interventions in emerging economies.

From 2012 to 2020, we empirically examine the relationship between technological innovation and corporate leverage using the financial data of A-share listed companies on China’s Shanghai and Shenzhen stock exchanges. The two variables have a significant negative correlation. By distinguishing further between the effects of policy incentives and market competition, we find that policy incentives have an “investment crowding out” effect. In contrast, market competition has an “innovation spillover” effect. These factors mitigate the negative relationship between technological innovation and corporate leverage. In addition, the relationship between the two is influenced by the nature of the firm, industry characteristics, location, and the firm’s life cycle.

Our research makes the following contributions: First, the relationship between technological innovation and corporate leverage is reconsidered. In contrast to answering the question of what types of corporations are conducive to innovation (He and Tian, 2013; Hsu et al., 2014; Cornaggia et al., 2015; Iqbal et al., 2022), the purpose of this study is to determine whether technological innovation can drive corporate leverage adjustment. Second, we investigate the effect of policy incentives and market competition on the relationship between technological innovation and corporate leverage. On this basis, we also observe regional differences to make the study more realistic. Thirdly, we contribute to the existing literature on corporate leverage by investigating the various effects of technological innovation on corporate leverage through cross-sectional, spatial, temporal, and leverage interval differentiation.

The remainder of the article is structured as follows: Section “Hypothesis development” is devoted to the formulation of hypotheses, Section “Data and methods” to the description of data and methods, Section “Analysis results” to the analysis results, Section “Robustness tests” to robustness tests, and Section “Conclusion” to the conclusion.

## Hypothesis development

Capital structure theory has become one of the most exciting areas of study in finance due to its unique insights into various issues about corporate leverage and firm value, as well as its vast and far-reaching implications for corporate financing decisions (Ma, 2021). It clarifies the significance of debt in the capital structure of a company. Companies no longer merely leverage users, but leverage designers, making selective and targeted adjustments to maximize company value and balance the internal and external environments.

Previous research on corporate leverage has generally focused on external (“taxation,” “bankruptcy”) and internal (“information transmission,” “agency costs”) factors, such as the cost of leverage adjustment, the soft budget constraint, the level of government control, the marketization process, policy influences, economic cycles, financial crises, and product market competition (MacKay and Phillips, 2005; Faulkender et al., 2012; Tsoy and Heshmati, 2017). However, the impact of technological innovation on corporate leverage has received scant attention.

### Technological innovation and corporate leverage

Early research on corporate leverage appeared unrelated to the firm’s value, as they did not consider the insolvency risk. Nevertheless, according to trade-off theory, during an economic downturn, financial crisis and bankruptcy risks force a firm’s market value into a paradox of “increased debt” and “financial risk” because of both its operating performance and the external environment (e.g., an infectious disease pandemic or loose monetary policy). A firm’s leverage can vary from its target capital structure. To maximize corporate value, additional adjustments to corporate leverage are necessary. Nonetheless, because the cost of adjustment exceeds its benefit, some businesses lack the incentive to leverage adjustment, which is detrimental to the interests of shareholders and severely restricts the development of the business (Fischer et al., 1989; Leary and Roberts, 2005; Ogawa, 2007; Strebulaev, 2007; Cook and Tang, 2010; Li et al., 2017). Consequently, it is necessary to elaborate on the factors driving the leverage adjustment.

In addition to influencing the sustainability of corporations, technological innovation integrates internal and external resources to spur environmental innovation (Horbach, 2008; Cai and Zhou, 2014). As a result, several academics have pondered whether technological innovation can facilitate corporate leverage adjustment. Some academics contend that technological innovation produces an innovative performance that drives rapid firm growth and has a tax shield effect that reduces financing costs. Therefore, technological innovation is having a deleveraging impact, which means that the more firms focus on innovation, the less debt they carry (Del Canto and Suarez Gonzalez, 1999; Graham, 2003; Graham and Tucker, 2006; Honore et al., 2015). Others contend that the opposite is true. They conclude that firms’ technological innovation capabilities are positively related to their debt levels because they are more willing to leverage and have higher debt levels because of the inherent consistency of their technological innovation goals, which reduce innovation risk and increase financial risk tolerance (David et al., 2000).

Indeed, technological innovation has two significant effects on corporate leverage: externally, by enhancing product market competitiveness, and internally, by strengthening corporate governance capabilities. These effects manifest themselves in sustained excess profits and industry-specific advantages. Because capital structure can reflect product market expectations of future competition, particularly for overleveraged firms, product market competition can force technological innovation and adjust leverage through monopoly rents and profits from intellectual property (e.g., patents) (Brander and Lewis, 1986; Showaher, 1995). In addition, technological innovation can help firms engage in capacity governance. These include creating differentiated new demand, transforming excess capacity into efficient capacity, and further optimizing distorted firm leverage by offsetting the cost of firm recapitalization with higher marginal product returns (Liu and Huang, 2016).

Consequently, based on the analysis presented above, we propose the following hypothesis:

**Hypothesis 1 (H1).** Technological innovation has a negative correlation with corporate leverage.

### Impact of policy incentives

Since technological innovation is a high-investment, long-cycle, and high-risk behavior, decision-makers neglect to invest in innovation when debt financing creates a constraint on free cash flow to maximize shareholders’ interests promptly (Jensen, 1986). In addition, because technological innovation involves patent protection and externality risks, corporations’ exogenous financing becomes extra cautious, discouraging R&D investment (Galende Del Canto and Suarez Gonzalez, 1999). Therefore, most technological innovation requires policy incentives.

Policy incentives are government interventions to redistribute resources in a market economy, avoiding value transfer caused by interest payments by reducing the cash consumption of technological innovation by businesses to stimulate technological innovation via direct or indirect policy assistance. Government subsidies, tax incentives, inter-institutional cooperation, and others are the primary policy incentives (Spence, 1984; David et al., 2000; Giudici and Paleari, 2000; Nishimura and Okamur, 2011). As China’s institutional development continues to advance, the government, as an innovation policymaker and strategist, is very concerned with fostering technological innovation in businesses by formulating pertinent policies. Nevertheless, due to the uncoordinated and uneven regional economic development, policy incentives in China may result in regional disparities.

Numerous studies have confirmed the correlation between policy incentives and the technological innovation of businesses. Positive studies indicate that policy incentives not only alleviate the financing constraints associated with technological innovation and effectively promote R&D in high-precision end technologies but also regulate firms’ cost, equity, and R&D budgets, with the potential to improve firms’ capital structure (Honore et al., 2015; Xia and Roper, 2016; Howell, 2017; Li et al., 2017; Wade, 2017; Chen et al., 2020). Consequently, in terms of the direct effects of policy incentives, firms that receive government assistance always receive more funding, greater tax benefits, an increase in innovation output, and an improvement in innovation performance in their innovation investments (Gonzalez et al., 2005). On the other hand, pessimistic studies contend that policy incentives can lead to a decline in the quality of corporate R&D, R&D investment crowding out, corruption and rent-seeking, and a distortion of the policy’s original intent (Belenzon and Berkovitz, 2010; Bernini and Pellegrini, 2011; Lee, 2011; Liu et al., 2019; Kong, 2020). Therefore, one cannot ignore the indirect effects of policy incentives.

First, policy incentives can have an “investment crowding out” effect on firms’ technological innovation. Firms may reduce their innovation investment to seek policy support and restructure their technological innovation projects, substituting long-term quality innovation with simple short-term innovation, resulting in the innovation of lower quality (Wallsten, 2000; Czarnitzki et al., 2011; Aghion et al., 2013; Stuart and Wang, 2016). Second, according to signaling theory, obtaining policy support can help firms send positive signals, causing investors to anticipate that the firm is undervalued and to readily attract lower-cost external financing, which may contribute to the increase in firm leverage (Takalo and Tanayama, 2010).

Based on the analysis presented above, we propose the following hypothesis:

**Hypothesis 2 (H2).** The negative relationship between technological innovation and corporate leverage is weakened by policy incentives.

### Impact of market competition

The study of firm leverage must pay close attention to the market competition of firms (Barton and Gordon, 1988; O’ Brien, 2003). Debt’s purpose is to give managers an incentive to manage more efficiently and reduce discretionary cash outlays that are not in the shareholders’ best interests. However, managers make decisions regarding leverage based on the firm’s competitive environment, which also reflects the firm’s industry (Williamson, 1963). As a result, competition can serve as an alternative to debt, limiting managers’ use of available cash flow. The greater the market competition, the greater the number of customer comparisons and options and the lower the firm’s excess returns. It is no longer advantageous for firms to increase their leverage, while it also exposes them to greater risk and uncertain outcomes (Williamson, 1975; Jensen and Meckling, 1976; Kohli and Jaworski, 1990; Botosan and Plumlee, 2005). With increased market competition, companies are consequently more cautious with leverage. It also suggests that firms tend to reduce their leverage as the market becomes more competitive, even without the influence of technological innovation.

In addition, because the external institutional environment influences technological innovation, market competition, a critical external governance mechanism, can be a good indicator of external environment changes (Torkkeli et al., 2019). According to the well-known “Schumpeter effect,” competition reduces firms’ excess profits, and monopoly is the most effective innovation driver. A high level of market competition increases the likelihood that innovation will be imitated or substituted, which tends to generate technological innovation spillover effects, resulting in a decline in innovation performance and discouraging firms from innovating. In other words, innovation declines as market competition rise (Schumpeter, 1942; Grossman and Helpman, 1993).

China’s reform and opening up have accelerated its marketization process. However, even though market mechanisms have been unleashed and have driven economic development, there is still the dilemma of significant differences in regional market competition and uncoordinated economic growth. On the one hand, regions with intense market competition exhibit a concentration of industry factors, resulting in “innovation spillover,” which deprives the private sector of the incentive to innovate. On the other hand, most of the leverage in China’s manufacturing sector is held by state-owned enterprises (SOEs), whose proximity to the government grants them access to exogenous financing, promoting higher leverage.

Considering the above analysis, market competition may dampen the relationship between technological innovation and corporate leverage. We propose the following hypothesis:

**Hypothesis 3 (H3).** The negative relationship between technological innovation and corporate leverage is weakened by market competition.

## Data and methods

### Data

Our initial sample consists of China’s A-share-listed manufacturing companies. The sample period spans 2012 through 2020. In addition, the China Stock Market & Accounting Research Database (CSMAR) and the East Money Data Platform provide information on the key independent variable, technological innovation (CHOICE). The remaining data comes from financial reports of publicly traded companies and the National Bureau of Statistics of China’s website.

The initial sample is screened based on the following four criteria: (1) the exclusion of companies designated as special treatment (ST) or special transfer (PT) during the sample period; (2) the removal of post-2012 listings; (3) the exclusion of companies with missing data during the sample period; and (4) the exclusion of companies with abnormal financial conditions, such as gross assets below zero and gearing below or above one. In addition, we eliminate the top and bottom 1% of each variable’s data set to avoid estimation bias. After completing the preceding steps, we obtained 8,841 valid sample observations.

### Variable definition

#### Corporate leverage

This study’s dependent variable is corporate leverage. Observing the change in corporate leverage reveals the adjustment pattern of corporate leverage. According to empirical studies, book value is favored over market value when discussing financial leverage (Stonehill et al., 1974). Therefore, corporate leverage is defined as the ratio of total debt to the book value of total assets.

#### Technological innovation

In this study, the independent variable is technological innovation. In this paper, technological innovation refers to technological innovation at the firm level, where a company creates a new product, offers a new service, or implements a new process. To reduce noise in measuring technological innovation, we introduce innovation intensity, which uses the ratio of R&D expenditures to operating revenue to measure corporations’ technological innovation (Nosheen et al., 2016).

#### Control variables

We control the size (Size), Return on Net Assets (ROE), Year-on-Year Growth Rate of Operating Income (Gro_oper), Financial Expenses (Debt), Liquidity of Assets (Liq_ass), and Corporate Age (Age) (Ramaswamy, 2001; Frank and Goyal, 2003; Mithas et al., 2012; Lusardi and Tufano, 2015; Klobucar and Orsag, 2019; Berger et al., 2022). The definitions and descriptive statistics for these variables are provided in Table 1.

**TABLE 1:** Definitions of the variables.

Variables	Definition
Lev	Total liabilities divided by total assets
R&D	R&D expenditure divided by operating income
Size	Take the natural log of total assets
ROE	Return on equity is measured by the ratio of net profit to net assets
Gro_oper	Operating income year-on-year growth rate in the previous period
Debt	Take the natural log of financial expenses
Liq_ass	Current assets divided by total assets
Age	Take the natural log of the years of establishment of the enterprise
Market	Marketization Index of China
Gover	Government subsidies divided by total assets
Scien	The natural log of regional science expenditure

#### Moderating variables

We utilized policy incentives and market competition to moderate the relationship between technological innovation and firm leverage. The China Marketization Index is used as a proxy variable for market competition, while government subsidies are used as a proxy variable for policy incentives. In place of specific government subsidy data, this study uses government subsidies in the income statements of publicly traded corporations divided by total assets (Jia et al., 2021). In addition, the China Marketization Index utilized in this paper is derived from Wang et al.’s (2019) “China Provincial Marketization Index Report,” which began in 1997 and has since published nine reports covering 31 provinces in China. These reports include government-market relations, the development of the non-state economy, the development of product markets, the development of factor markets, the development of market intermediary organizations, and the rule of law environment. It consists of seventeen fundamental indices in five areas and is a comprehensive index that can more accurately reflect marketization. As the index is only updated to 2019, the data for this analysis spans 2012 through 2019.

### Methods

The following empirical model was developed to examine the impact of technological innovation on corporate leverage.


(1) $$L\,{\text{ev}}_{i,t}=\alpha +\beta \times R\&D_{i,t}+\theta \times C\,o\,n\,t\,r\,o\,l_{i,t}+\omega_{i}+\delta_{t}+\xi_{i,t}$$

The subscripts i and t represent firms and years, respectively. Lev_*i,t*_ represents corporate leverage while R&D_*i,t*_ represents innovation intensity. Control_*i,t*_ represent the multiple control variables. In this study, we also control for year fixed effectsδ_*t*_ and firm fixed effects δ_*i*_. To investigate further the impact of policy incentives and market competition on the relationship between technological innovation and corporate leverage, we augment models (2) and (3) with interaction terms.


$$L\,{\text{ev}}_{i,t}=\alpha_{0}+\alpha_{1}\times R\&D_{i,t}+\beta_{1}$$

$$\times G\,{\text{over}}_{i,t}+\beta_{2}\times R\&D_{i,t}\times G\,{\text{over}}_{i,t}+\theta$$

(2) $$\times C\,o\,n\,t\,r\,o\,l_{i,t}+\omega_{i}+\delta_{t}+\xi_{i,t}$$

$$L\,{\text{ev}}_{i,t}=\alpha_{0}+\alpha_{1}\times R\&D_{i,t}+\beta_{1}\times {\text{Market}}_{i,t}+\beta_{2}\times R\&D_{i,t}$$

(3) $$\times {\text{Market}}_{i,t}+\theta \times C\,o\,n\,t\,r\,o\,l_{i,t}+\omega_{i}+\delta_{t}+\xi_{i,t}$$

Gover_*i,t*_ and Market_*i,t*_ are measures of policy incentives and market competition, R&D_*i,t*_ × Gover_*i,t*_ denotes the moderating effect of policy incentives on the relationship between technological innovation and corporate leverage, and R&D_*i,t*_ × Market_*i,t*_ denotes the moderating effect of market competition on the relationship between technological innovation and corporate leverage.

## Analysis results

### Statistical analysis

#### Descriptive statistics

Table 2 displays descriptive statistics for the most critical variables. Based on the average leverage value, Chinese manufacturing companies’ average annual leverage ratio was 0.401. Regarding the leverage structure, short-term leverage accounted for 83.8%, and long-term leverage accounted for 16.2%, indicating that the sample manufacturing enterprises’ debt financing was primarily short-term. The range of technological innovation values is from 0.001 to 0.198, with a mean, standard deviation, and median of 0.042, 0.029, and 0.037, respectively, indicating that corporations vary widely in terms of innovation intensity, but the majority are also incompetent. Also included in Table 2 are summary statistics for our control variables.

**TABLE 2:** Descriptive statistics of the variables.

	*N*	Mean	SD	Skewness	Kurtosis	P25	P50	P75	Min	Max
Lev	8,841	0.401	0.174	–0.828	0.126	0.262	0.397	0.533	0.055	0.852
Shor_lev	8,841	0.336	0.151	–0.631	0.279	0.215	0.327	0.442	0.043	0.755
Lon_lev	8,841	0.065	0.066	1.017	1.289	0.014	0.039	0.1	0	0.314
R&D	8,841	0.042	0.029	4.46	1.689	0.025	0.037	0.052	0.001	0.198
Size	8,841	8.378	1.066	0.01	0.573	7.61	8.261	8.997	6.287	11.81
ROE	8,841	0.068	0.091	–1.163	8.905	0.027	0.065	0.114	–0.483	0.353
Gro_oper	8,841	0.113	0.222	2.421	0.979	–0.022	0.089	0.215	–0.444	1.22
Debt	8,841	0.007	0.01	–0.076	0.382	0	0.006	0.013	–0.017	0.036
Liq_ass	8,841	0.569	0.149	–0.674	–0.135	0.46	0.573	0.684	0.195	0.89
Age	8,841	2.871	0.303	1.511	–0.415	2.708	2.89	3.045	0.693	4.174
Market	7,833	9.410	1.465	–1.103	4.240	8.607	9.656	10.560	3.359	11.494
Gover	8,562	0.006	0.006	6.472	95.412	0.002	0.004	0.007	0	0.154

Table 1 reports the summary statistics of key variables.

#### Correlation coefficient test

The correlation coefficient between corporate technical innovation and corporate leverage is negative, as demonstrated in Table 3.

**TABLE 3:** Pearson correlation coefficients of the variables.

	Lev	R&D	Size	ROE	Gro_oper	Debt	Liq_ass	Age	Market	Gover
Lev	1									
R&D	−0.239**	1								
Size	0.499**	−0.187**	1							
ROE	−0.168**	−0.059**	0.108**	1						
Gro_oper	−0.025*	0.018	0.002	0.296**	1					
Debt	0.673**	−0.214**	0.241**	−0.244**	−0.064**	1				
Liq_ass	−0.114**	0.112**	−0.142**	0.127**	0.024*	−0.343**	1			
Age	0.113**	−0.067**	0.218**	−0.037**	−0.089**	0.074**	−0.089**	1		
Market	−0.073**	0.092**	−0.040**	0.022*	0.019	−0.075**	0.008	0.168**	1	
Gover	0.382**	0.010	0.701**	0.091**	–0.013	0.184**	−0.126**	0.172**	−0.015	1

*Statistical significance at the 10 % level, **statistical significance at the 5% level, ***statistical significance at the 1% level.

### Baseline result

The impact of technological innovation on corporate leverage is examined in Table 4. Column (1) does not include control variables. The coefficient on technological innovation is negative and statistically significant (–0.596, *p*-value < 0.01), according to the regression results. In column (2), which contains the independent variables, the technological innovation coefficient remains negative and statistically significant (–0.412, *p*-value < 0.01). Our findings concur with national studies (Singh and Faircloth, 2005; Min and Smyth, 2016). It supports H1, in which technological innovation correlates negatively with corporate leverage.

**TABLE 4:** Impact of technological innovation on corporate leverage.

Dependent variable	Lev (1)	Lev (2)
R&D	–0.596***	–0.412***
	(–5.24)	(–4.71)
Size		0.070***
		(14.42)
Age		0.056**
		(1.98)
ROE		–0.100***
		(–6.57)
Gro_oper		0.028***
		(6.19)
Debt		7.255***
		(29.95)
Liq_ass		–0.006
		(–0.31)
Constant		–0.341***
		(–3.95)
Industry FE	Yes	Yes
Year FE	Yes	Yes
*N*	8,841	8,841
Within *R*-sq	0.0477	0.4285

*Statistical significance at the 10 % level, **statistical significance at the 5% level, ***statistical significance at the 1% level. *T*-value are reported in parentheses.

First, the tax shield effect of leverage provides a broader scope for technological innovation and cheaper financial support through profitability signals (Brown and Petersen, 2011). Second, technological innovation increases firms’ productivity and facilitates their rapid growth. Third, it increases the industry’s competitiveness and market share and can motivate the firm to adjust its leverage to achieve its desired capital structure (Ciftci and Cready, 2011). Fourth, incentives for technological innovation can also reduce firms’ innovation expenses and boost their profitability (Czarnitzki and Licht, 2006; Hewitt-Dundas and Roper, 2010). Lastly, from the perspective of the relationship between excess capacity and distortionary leverage, technological innovation assists firms in governing their capacity, enables them to increase profits despite excess capacity, and shifts their leverage toward an optimal band (Almeida and Campello, 2007; Yu, 2017).

### Impact of policy incentives

The effect of policy incentives on the relationship between technological innovation and corporate leverage is then investigated. In column (1) of Table 5, the effects of policy incentives are detailed. Before constructing the interaction terms, all variables are centralized to prevent multicollinearity issues. Column (1) demonstrates that the coefficient on technological innovation is significantly negative at the 1% level. In comparison, the coefficient on the interaction term between government subsidies and innovation intensity is significantly positive at the 5% level, suggesting that policy incentives have a dampening effect on them, which supports the hypothesis of H2 that policy incentives weaken the negative relationship between technological innovation and corporate leverage.

**TABLE 5:** Impact of policy incentives and market competition on the relationship between technological innovation and corporate leverage.

Dependent variable	Lev (1)	Lev (2)
R&D	–0.502***	–1.087**
	(–5.11)	(–2.48)
Gover	–0.439	
	(–1.48)	
Gover*R&D	0.119**	
	(2.36)	
Market		0.006*
		(1.80)
Market*R&D		0.077*
		(1.69)
Control variables	Yes	Yes
Constant	–0.323***	–0.357***
	(–3.67)	(–3.76)
Industry FE	Yes	Yes
Year FE	Yes	Yes
*N*	8,562	7,833
Within *R*-sq	0.4268	0.4222

***Indicates significance at the 1% level, **indicates significance at the 5% level, and *indicates significance at the 10% level.

The possible explanation is that, contrary to positive intuition, while the direct effect of policy incentives makes firms more innovative. The indirect effect of policy incentives also has an “investment crowding out” effect on firms’ technological innovation. As observed, China’s manufacturing sector is more consistent with the “investment crowding out” effect. In previous studies, Liu et al. (2019) suggested the need for a more targeted use of government subsidies to incentivise firms to innovate.

### Impact of market competition

To further examine the relationship between technological innovation and firm leverage, we incorporate market competition into the model. Column 2 of Table 5 reported the moderating effects of market competition. Controlling for other variables, the coefficient on technological innovation is significantly negative at the 5% level. In contrast, the coefficient on the interaction term between technological innovation and market competition is significantly positive at the 10% level, indicating that market competition mitigates the negative relationship between technological innovation and corporate leverage. H3 is supported, i.e., market competition mitigates the negative relationship between technological innovation and corporate leverage.

The result can be explained by market competition’s “innovation spillover” effect. Specifically, the involuntary welfare spillover to peers and society, for which there is no return, undermines firms’ incentive to innovate. However, it should also be noted that in highly competitive markets, excessive competition can reduce firms’ excess profits, resulting in a greater emphasis on survival and a selective reduction in firms’ willingness to take risks and incentives to innovate (Schumpeter, 1942; Xie and Wei, 2016). Consequently, the impact of technological innovation on firms’ deleveraging can be moderated by market competition and is variable.

### Further analysis

To observe additional regional heterogeneity in the moderating effect, we refer to the division of economic regions in the Opinions of the Central Committee of the Communist Party of China and the State Council on Promoting the Rise of the Central Region and the Opinions of the State Council on the Implementation of Certain Policy Measures for the Development of the Western Region and divide them into three major economic regions for group regression: East, West and Central. The classifications are as follows: East: Beijing, Fujian, Guangdong, Hainan, Hebei, Jiangsu, Shandong, Shanghai, Tianjin, Zhejiang, Liaoning, Jilin, Heilongjiang; Central: Anhui, Henan, Hubei, Hunan, Jiangxi, Shanxi; West: Gansu, Guangxi, Guizhou, Inner Mongolia, Ningxia, Qinghai, Shaanxi, Sichuan, Tibet, Xinjiang, Yunnan, Chongqing.

The results of regional differentiation for the effects of policy incentives and market competition are presented in Tables 6, 7, respectively. The estimated results of the baseline regression are presented in columns (1), (2), and (3). Meanwhile, the regression of the moderating effects is presented in columns (4), (5), and (6). The coefficient on R&D in column (1) of Table 6 for the eastern region is significantly negative and statistically significant (–0.478, *p*-value < 0.01), indicating that technological innovation in the eastern region can effectively contribute to corporate deleveraging. The coefficient on Gover*R&D is statistically significant and significantly positive in column (4) of Table 6 (0.154, *p*-value < 0.1), and the coefficient on Market*R&D is statistically significant and significantly positive in column (4) of Table 7 (0.152, *p*-value < 0.05). It demonstrates that the east’s policy incentives and market competition mitigate the negative relationship between technological innovation and corporate leverage. The coefficient on R&D in column (3) of Table 6 is negative but not statistically significant; the coefficient on Gover*R&D in column (6) of Table 6 is significantly positive and statistically significant (0.343, *p*-value < 0.01), and the coefficient on Market*R&D in column (7) of Table 7 is significantly positive and statistically significant (0.266, *p*-value < 0.05). In other words, policy incentives and market competition dampen the technology innovation-corporate leverage relationship in the western region. The central region lacks a significant moderating effect.

**TABLE 6:** A comparison of regression results on the relationship between technological innovation and corporate leverage in different regions before and after the inclusion of policy incentives.

Variables	(1) Lev(E)	(2) Lev(C)	(3) Lev(W)	(4) Lev(E)	(5) Lev(C)	(6) Lev(W)
R&D	–0.478***	–0.200	–0.312	–0.581***	–0.162	–0.559*
	(–4.48)	(–1.15)	(–1.07)	(–4.88)	(–0.79)	(–1.95)
Gover				–0.553	0.211	–1.840**
				(–1.44)	(0.29)	(–2.30)
Gover*R&D				0.154*	–0.046	0.343***
				(1.91)	(–0.53)	(3.46)
Control variables	Yes	Yes	Yes	Yes	Yes	Yes
Constant	–0.396***	0.132	–0.435	–0.361***	0.119	–0.492
	(–3.92)	(0.74)	(–1.51)	(–3.55)	(0.66)	(–1.54)
Industry FE	Yes	Yes	Yes	Yes	Yes	Yes
Year FE	Yes	Yes	Yes	Yes	Yes	Yes
*N*	6,301	1,495	1,045	6,096	1,464	1,002
Within *R*-sq	0.4456	0.3776	0.4355	0.4460	0.3722	0.4280

Dependent variable: Lev(E) represents manufacturing companies in the East, Lev(C) represents Manufacturing companies in the Central, Lev(W) represents Manufacturing companies in the West. This table reports the changes in the relationship between technological innovation and corporate leverage for different regions before and after the inclusion of policy incentives.

*T*-values are reported in parentheses l.

***Statistical significance at the 1% level.

**Statistical significance at the 5% level.

*Statistical significance at the 10% level.

**TABLE 7:** A comparison of regression results on the relationship between technological innovation and corporate leverage in different regions before and after the inclusion of market competition.

Variables	(1) Lev(E)	(2) Lev(C)	(3) Lev(W)	(4) Lev(E)	(5) Lev(C)	(6) Lev(W)
R&D	–0.478***	–0.200	–0.312	–1.952***	1.755	–2.161**
	(–4.48)	(–1.15)	(–1.07)	(–3.21)	(1.08)	(–2.24)
Market				0.001	0.006	0.013
				(0.32)	(0.41)	(1.28)
Market*R&D				0.152**	–0.222	0.266**
				(2.49)	(–1.20)	(2.01)
Control variables	Yes	Yes	Yes	Yes	Yes	Yes
Constant	–0.396***	0.132	–0.435	–0.365***	–0.044	–0.293
	(–3.92)	(0.74)	(–1.51)	(–3.21)	(–0.18)	(–1.13)
Industry FE	Yes	Yes	Yes	Yes	Yes	Yes
Year FE	Yes	Yes	Yes	Yes	Yes	Yes
*N*	6,301	1,495	1,045	5,600	1,325	908
Within *R*-sq	0.4456	0.3776	0.4355	0.4422	0.3634	0.4377

Dependent variable: Lev(E) represents companies in the East, Lev(C) represents companies in the Central, Lev(W) represents companies in the West. This table reports the changes in the relationship between technological innovation and corporate leverage for different regions before and after the inclusion of market competition. *T*-values are reported in parentheses l.

***Statistical significance at the 1% level.

**Statistical significance at the 5% level.

*Statistical significance at the 10% level.

In contrast to the negative relationship between technology innovation and corporate leverage in the benchmark regression, the coefficients on Gover*R&D and Market*R&D are significantly positive for both the eastern and western regions when the effects of policy incentives and market competition are accounted for. In addition, when both are present, the deleveraging effect of technological innovation in the western region changes from negligible to significant. Possible causes include the injection of political and economic dynamism into China’s western regions through strategies such as developing the west and revitalizing the countryside. In addition, it can effectively facilitate access to innovation support (e.g., policies, resources, technology) and cheaper funding for innovation projects (e.g., government grants, loans for inclusive finance projects) for businesses in the west. Therefore, it is implicitly a policy recommendation that continued improvements in the institutional and economic environment are more conducive to exploiting the adverse effects of technological innovation on corporate leverage.

Lastly, the insignificance of the central region may be due to the unbalanced development strategies implemented in China, such as priority development in the east, development in the west, and revitalization of old industrial bases, which have objectively created a “depression” in the macroeconomic pattern of the central region. It results in the total economic development of the central region being lower than that of the eastern region, and the growth rate of the central region is slower than that of the eastern region. As a result, the total economic development of the central region is lower than that of the eastern region, and the growth rate of economic development in the central region is lower than that of the western region.

## Robustness tests

In this section, we conduct several checks on the reliability of our key findings.

### IV regression results

We chose science expenditure as the instrumental variable to address the potential endogeneity issue. There are reasons for the selection of instrumental variables. Firstly, as regional science expenditure is macro-policy support, unlike profit-seeking firms on the market, the government intends to optimize the regional structure and will provide support to firms across the region without an explicit preference for supporting certain firms. Second, when GDP is the primary metric for measuring political performance, governments have a self-interested investment preference to favor production over innovation due to innovation’s long-term and unpredictable nature, resulting in a high degree of regional variation in science spending. Lastly, regional science expenditure typically reflects the level of support for innovation and the conditions for innovation that the region can provide to firms. It is closely related to the paper’s subject’s technological innovation. Table 8 displays the results of the two-stage least squares regression for the IV method, which indicates that technological innovation has a negative regression coefficient. The test results for the weak instrumental variables indicate that the F-statistic for the first stage is 65.29, which is well above the critical value of 10 for the weak instrumental variables (Hausman et al., 2005).

**TABLE 8:** The results of the instrumental variables approach to regression.

Dependent variables	Lev
R&D	–1.256**
	(–2.49)
Control variables	Yes
Constant	–0.201***
	(–4.98)
Industry FE	Yes
Year FE	Yes
**First-stage regression**	
IV	0.003***
	(8.12)
*N*	8,841
Adj *R*-sq	0.1715
Robust F	65.92
Prob > F	0.000

This table presents IV approach results that use Government Science Expenditure as an instrumental variable. *T*-values are reported in parentheses. ***Indicates significance at the 1% level, **indicates significance at the 5% level, *indicates significance at the 10% level.

### Different model designs

We test the robustness of the model by substituting the proxy variables for technological innovation with one lag of technological innovation (L.R&D), one period of R&D expenditure taking the natural logarithm (Innov), and one period of lag R&D expenditure (L.Innov) while maintaining the original control variables. The regression results are in columns (1) through (3) of Table 9. After replacing the core explanatory variables in the benchmark model with each of the three new independent variables, we find that the coefficient on L.R&D is significantly negative at the 1% significance level in the two-fixed effects panel regression. At the same time, the coefficients on Innov and L.Innov are also significantly negative at the 5% significance level, respectively. In this instance, the negative effect of technological innovation on firm leverage is once more demonstrated.

**TABLE 9:** The results of different models and variables.

Dependent variables	Lev (1)	Lev (2)	Lev (3)
L.R&D	–0.403***		
	(–4.42)		
Innov		–0.007**	
		(–2.12)	
L.Innov			–0.007**
			(–2.30)
Control variables	Yes	Yes	Yes
Constant	–0.399***	–0.379***	–0.434***
	(–3.70)	(–4.34)	(–3.97)
Industry FE	Yes	Yes	Yes
Year FE	Yes	Yes	Yes
*N*	7,319	8,841	7,319
Within *R*-sq	0.3856	0.4256	0.3829

This table presents the results of a robustness test of different model designs and variables.

*T*-values are reported in parentheses. ***Indicates significance at the 1% level, **indicates significance at the 5% level, *indicates significance at the 10% level.

### Cross-sectional heterogeneity test

#### Different ownership

Table 10 shows the outcomes of grouped regressions by ownership and industry to determine under what conditions the impact of technological innovation on firm leverage would increase. Column (1) is for SOEs with a statistically significant negative coefficient on R&D (–0.476, *p*-value < 0.01); column (2) is for non-SOEs, with a statistically significant negative coefficient on R&D (–0.378, *p*-value < 0.01). (|–0.476| >|–0.378|) demonstrates that technological innovation negatively affects SOE leverage.

**TABLE 10:** Cross-sectional analysis of ownership status and industry effects.

Dependent variables	(1) Lev(S)	(2) Lev(N)	(3) Lev(H)	(4) Lev(T)
R&D	–0.476***	–0.378***	–0.568***	–0.338***
	(–3.08)	(–3.59)	(–4.94)	(–2.80)
Control variables	Yes	Yes	Yes	Yes
Constant	–0.430**	–0.291***	–0.553***	–0.246**
	(–2.49)	(–2.94)	(–3.96)	(–2.37)
Industry FE	Yes	Yes	Yes	Yes
Year FE	Yes	Yes	Yes	Yes
*N*	2,728	6,113	2,015	6,826
Within *R*-sq	0.3942	0.4435	0.4703	0.4241

Dependent variable: Lev(S) represents state-owned enterprises, Lev(N) represents non-state-owned enterprises, Lev(H) represents high tech enterprises; Lev(T) represents traditional enterprises. ***Indicates significance at the 1% level, **indicates significance at the 5% level, and *indicates significance at the 10% level.

Two-thirds of the leverage in China’s non-financial sector is held by SOEs, and the leverage ratio of SOEs is growing faster than that of private enterprises, giving them more room for downward adjustment. Additionally, SOEs have stronger incentives and conditions for technological innovation because they must transform their production processes and optimize their production capacity through innovation. In addition, the Chinese government has advocated for the active participation of SOEs in technological innovation, resulting in an external effect-induced skewing of SOEs’ policies.

#### Different industries

Table 10, columns (3) and (4) display the results of regressions grouped by industry characteristics. The coefficient on R&D in high-tech industries is negative and statistically significant (–0.568, *p*-value < 0.01); the coefficient on R&D in traditional manufacturing industries is also negative and statistically significant (–0.338, *p*-value < 0.01). Our comparison reveals that the effect of technological innovation on deleveraging is significantly greater in high-tech industries than in traditional industries.

This difference may be attributed to the knowledge-intensive nature of high-tech industries, which necessitates efficient innovation for firms to gain market share rapidly. Despite the fiercer market competition, high-tech firms’ high innovation capacity can reduce innovation costs. As a result, decision-makers are more likely to invest more resources in innovation, rapidly increasing technological innovation and enhancing its deleveraging effect.

### Different firm lifecycle stages

Our lifecycle classification is based on Dickinson’s cash flow-based method for determining the stage of a company’s lifecycle (Dickinson, 2006). In Table 11, we present the regression results for three different lifecycle firms to investigate the firm’s lifecycle impact. (The start-up period is excluded from the test because we are examining publicly traded corporations). In columns (1), (2), and (3), the coefficient on R&D is significantly negative and statistically significant (–0.318, *p*-value < 0.01), (–0.546, *p*-value < 0.01) and (–0.537, *p*-value < 0.01). It indicates that the negative effect of technological innovation on firm leverage persists regardless of the firm’s growth, maturity, or recessionary status. In |–0.546| >|–0.537| >|–0.318|, the period of maturity has the greatest effect, followed by the period of recession, and the period of growth has the least.

**TABLE 11:** Impact analysis of business life cycle.

Dependent variables	(1) Lev(G)	(2) Lev(M)	(3) Lev(R)
R&D	–0.318***	–0.546***	–0.537***
	(–2.68)	(–4.67)	(–2.98)
Control variables	Yes	Yes	Yes
Constant	–0.133	–0.352***	–0.833***
	(–1.00)	(–3.51)	(–4.16)
Industry FE	Yes	Yes	Yes
Year FE	Yes	Yes	Yes
*N*	3,832	3,552	1,457
Within *R*-sq	0.3998	0.4727	0.5203

Dependent variable: Lev(G) represents growth stage enterprises, Lev(M) represents mature enterprises; Lev(R) represents recession enterprises. ***Indicates significance at the 1% level, **indicates significance at the 5% level, and *indicates significance at the 10% level 4.

It may be because mature firms have greater profitability and market competitiveness, preparing them for deleveraging. In addition, firms with increasingly stable innovation resources can effectively increase their innovation success at a lower innovation cost, resulting in the greatest deleveraging effect of technological innovation on mature-stage businesses. In addition, possessing increasingly stable innovation resources can reduce innovation costs and increase a company’s innovation success rate. Therefore, firms can achieve the greatest deleveraging effect of technological innovation at the mature stage.

### Different leverage intervals

In Table 12, we present the regression results for the low and high-leverage intervals to investigate the effect of technological innovation on the various leverage intervals. The R&D coefficients in columns (1) and (2) are both significantly negative and statistically significant (–0.337, *p*-value < 0.01) and (–0.212, *p*-value < 0.1). It indicates that low and high-leverage firms can deleverage through technological innovation, further supporting our findings.

**TABLE 12:** Impact analysis of different leverage intervals.

Dependent variables	(1) Lev(low)	(2) Lev(high)
R&D	–0.337***	–0.212*
	(–4.05)	(–1.65)
Control variables	Yes	Yes
Constant	–0.124*	–0.00004
	(–1.67)	(–0.00)
Industry FE	Yes	Yes
Year FE	Yes	Yes
*N*	4,493	4,348
Within *R*-sq	0.3323	0.2678

Lev(low) refers to the sample of firms with leverage less than or equal to the mean (0.401); Lev(high) refers to the sample of firms with leverage greater than the mean (0.401).

***Indicates significance at the 1% level, **indicates significance at the 5% level, *indicates significance at the 10% level.

## Conclusion

Due to its capital-related nature, corporate leverage is highly exposed to financial risk, and optimizing corporate leverage is an effective method of mitigating financial risk. We present new evidence on the relationship between technological innovation and corporate leverage using China-specific data. Unlike previous research, this study investigates technological innovation while considering the effects of policy incentives and market competition. The relationship between technological innovation and corporate leverage is generally negative. Simultaneously, they exhibit significant cross-sectional, spatial, and temporal heterogeneity. Furthermore, policy incentives produce an “investment crowding out” effect, whereas market competition produces an “innovation spillover” effect. Both factors inhibit the relationship between technological innovation and corporate leverage. Further differentiating by region, it is observed that both policy incentives and market competition in the eastern and western regions can have a significant negative moderating effect on the relationship between technology innovation and corporate leverage.

The effectiveness of China’s Structural Deleveraging Policy and National Innovation Strategy is reflected in our analysis. Technological innovation plays a significant role in the adjustment of corporate leverage. By encouraging firms to engage in technological innovation, policymakers in emerging economies can maximize corporate leverage levels. The heterogeneity tests also indicate that by actively guiding state-owned firms, high-technology firms, and mature firms toward technological innovation, governments can improve the efficiency of firms’ leverage adjustment, increase their investment in innovation, and decrease their reliance on low-end products. Moreover, continued focus on and improvement of the policy, investment, and legal environments can better leverage the moderating effect of technological innovation on corporate leverage.

Our study has some limitations. In the first place, when measuring technological innovation, we have only considered innovation inputs. Other factors, such as innovation output and efficiency, should also be considered. Second, due to our reliance on financial data, we have yet to measure the regional impact of technological innovation. Future research will expand the measurement of spatial spillover effects. In addition, the response of firm leverage to technological innovation is influenced by various factors, such as financial inclusion and emergencies, all of which merit further investigation in future studies.

## Data availability statement

The original data supporting the conclusions of this article will be provided by the corresponding author.

## Author contributions

DX developed the theoretical framework. DL worked on data analysis. LR worked on literature review and manuscript writing. All authors contributed to the article and approved the submitted version.
